# Complete Genome Sequence of a Novel Natural Recombinant Porcine Reproductive and Respiratory Syndrome Virus Isolated from a Pig Farm in Yunnan Province, Southwest China

**DOI:** 10.1128/genomeA.00003-12

**Published:** 2013-01-24

**Authors:** Yulin Yan, Aiguo Xin, Gaohong Zhu, Hui Huang, Qian Liu, Zhiyong Shao, Yating Zang, Ling Chen, Yongke Sun, Hong Gao

**Affiliations:** a Faculty of Animal Science and Technology, Yunnan Agricultural University, Kunming, Yunnan, China; b Yunnan Animal Science and Veterinary Institute, Kunming, Yunnan, China; c Department of Nuclear Medicine, the First Affiliated Hospital of Kunming Medical University, Kunming, Yunnan, China

## Abstract

YN-2011 is a highly pathogenic North American porcine reproductive and respiratory syndrome virus (PRRSV). Unlike previously described PRRSVs, which contained a 30-amino-acid deletion in NS2, YN-2011 had no amino acid deletions or insertions but had several new mutations in NS2. Here, we announce the complete genome sequence of YN-2011.

## GENOME ANNOUNCEMENT

Porcine reproductive and respiratory syndrome virus (PRRSV) is the cause of an economically important swine disease that has been devastating the swine industry since the late 1980s (1). PRRSV is a positive-strand RNA virus belonging to the genus *Arterivirus* of the family *Arteriviridae* in the order *Nidovirales* (1–4). Since its appearance in southern China in 2006, highly pathogenic porcine reproductive and respiratory syndrome virus (HP-PRRSV) has the characteristic of a discontinuous 30-amino-acid (aa) deletion in nonstructural protein 2 (NS2) (5, 6). Under the strong immune pressures caused by the current control strategy, PRRSVs are geared for rapid variation through mutation or recombination (7–9), resulting in new isolates with different levels of pathogenicity and virulence. In this study, we report a complete genomic sequence of the local novel natural recombinant PRRSV, characterized by the lack of the NS2 deletion, but which also can cause serious clinical symptoms, including death, which were isolated from vaccinated pig farms in the Yunnan province of southwest China.

Serum and tissue samples were collected from growing pigs suspected to be infected in the Yunnan Province in 2011. The infected pigs were about 10 weeks old and displayed symptoms similar to those of original PRRSV, including severe respiratory distress, emaciation, and depression. Furthermore, this disease resulted in a 5.6% mortality rate on this farm. A PRRSV field isolate, named YN-2011, was propagated on Marc-145 cells, and the total viral RNA was extracted from the lung tissue and infected cell culture and then was used separately for the amplification of the genome. Basically, 18 primer pairs were used to generate overlapping amplicons by reverse transcription PCR (RT-PCR). The PCR products were cloned into pMD19T vector (TaKaRa), sequenced three times using an ABI 3730 Sanger-based genetic analyzer, and assembled using DNAStar version 7.1 to obtain the complete genome sequence. The completed sequence showed that, including the poly(A) tail, the genomic sequence of YN-2011 was 15,432 nucleotides in length. Genetic analyses demonstrated that YN-2011 shared 99.5% genome similarity with strain VR-2332, but a dramatically low degree of genetic homology (37.2%) with the representative highly pathogenic PRRSV JXA1 strain was observed; the phylogenetic tree showed that YN-2011 and VR-2332 are the same subgroup. The NS2 of YN-2011 and that of the VR-2332 strain show a genetic homology of 99.4%. There is no amino acid deletion or insertion in NS2 protein sequence, but there are new several substitutions, such as T-I (111), S–F (285), EV-QL (345, 346), P–S (475), T-A (491), Y–H (566), D–N (568), H–R (600), R–H (684), M-K (768), and F-L (926) (amino acid positions are given in parentheses).

In contrast with the recombinant or overattenuated HP–PRRSV strain as has been reported previously (5, 9, 10), the YN-2011 strain could induce typical clinical symptoms. However, the results identify the viewpoint that the 30-amino-acid (aa) deletion in NS2 is not related to the virulence of the virus (11). Our study indicates that the HP–PRRSV variant, regardless of the 30-aa deletion, continues to have a prevailing and accelerating evolution in China. The present finding of the YN-2011 sequence will enhance our understanding not only of the PRRSV evolutionary mechanism, but also of how to control PRRS disease.

### Nucleotide sequence accession number.

The virus genome sequence of strain YN-2011 is available in GenBank under accession no. JX857698.
