# Complete Genome Sequence of a Novel Natural Recombinant Porcine Reproductive and Respiratory Syndrome Virus Isolated from a Pig Farm in Yunnan Province, Southwest China

**DOI:** 10.1128/genomeA.00101-13

**Published:** 2013-03-07

**Authors:** Yulin Yan, Aiguo Xin, Gaohong Zhu, Hui Huang, Qian Liu, Zhiyong Shao, Yating Zang, Ling Chen, Yongke Sun, Hong Gao

**Affiliations:** Faculty of Animal Science and Technology, Yunnan Agricultural University, Kunming, Yunnan, China; Yunnan Animal Science and Veterinary Institute, Kunming, Yunnan, China; Department of Nuclear Medicine, the First Affiliated Hospital of Kunming Medical University, Kunming, Yunnan, China

## ERRATUM

Volume 1, no. 1, e00003-12, 2013. Page 1: NS2 should read Nsp2 throughout the abstract and text.

